# Saliva as a window into tumor immunity in oral squamous cell carcinoma: tissue concordance and translational validation

**DOI:** 10.3389/fimmu.2026.1957454

**Published:** 2026-10-08

**Authors:** Huiting Hu, Yong Yang

**Affiliations:** 1 Department of Oral Medicine, Stomatological Hospital, Southern Medical University, Guangzhou, China; 2 Department of Periodontology and Oral Implantology, Stomatological Hospital, Southern Medical University, Guangzhou, China

**Keywords:** immune biomarkers, liquid biopsy, oral squamous cell carcinoma, saliva, tissue concordance, translational validation, tumor immune microenvironment

## Abstract

Saliva is non-invasive, repeatable, and anatomically close to oral squamous cell carcinoma (OSCC), but proximity does not establish that a salivary biomolecule originates from the tumor. Cytokines, extracellular vesicles, coding and non-coding RNAs, and tumor-derived DNA may arise from tumor and immune cells, adjacent mucosa, periodontal tissues, salivary glands, oral microbiota, or systemic circulation. This review evaluates whether salivary immune signals reflect the OSCC tissue immune microenvironment using three evidence levels: quantitative paired tissue-saliva concordance within patients (Level I); indirect clinicopathological, source-enriched, paired-biofluid, or longitudinal support without quantitative paired tissue analysis (Level II); and saliva-only case-control differences (Level III). IL-6, IL-8/CXCL8, IL-1β, and TNF-α show relatively consistent salivary changes, whereas evidence for IL-10, selected chemokines, extracellular vesicle-associated PD-L1, and secreted miRNAs or lncRNAs remains limited or inconsistent. Salivary tumor DNA can show strong tissue-genotype concordance, but reflects tumor-derived material rather than immune state. Most immune candidates remain at Level II or III; none robustly meets Level I. Periodontitis, mucositis, infection, smoking, tumor ulceration, salivary flow, and sample processing complicate interpretation. We propose paired collection of tumor, adjacent mucosa, saliva, and plasma; spatial immunophenotyping; oral-inflammation controls; longitudinal perioperative and treatment sampling; standardized assays; and multicenter validation. The framework prioritizes biological traceability over diagnostic discrimination. At present, saliva is best considered a complementary longitudinal biosample, not a substitute for tissue immunophenotyping.

## 1 Introduction

OSCC constitutes most oral malignancies and remains burdensome, particularly after lymph-node metastasis (1, 2). Clinical examination and biopsy are indispensable but unsuitable for repeated monitoring. Immune-checkpoint inhibitors treat recurrent or metastatic HNSCC, yet benefit varies and assessment generally relies on tissue PD-L1 combined positive score rather than a non-invasive dynamic readout (3). Spatial studies show that cell counts alone miss immune status because malignant, immune, stromal, and endothelial cells occupy distinct neighborhoods (4). Saliva is inexpensive, repeatable, and close to oral tumors. Case-control studies report elevated salivary cytokines and proteins, whereas EV and nucleic-acid evidence often comes from small, platform-dependent studies (5, 6). A 2024 network meta-analysis of 40 oral-cancer studies found higher IL-6, IL-8, IL-10, TNF-α, and IL-1β but identified confounding, non-standardized methods, and inadequate samples (7). Reviews including OPMD or exposure-related risks show that diagnostic performance depends on cohort definition, with limited evidence for prognosis or treatment monitoring (5).

The central uncertainty is signal origin. Elevated cytokines may derive from tumor secretion, myeloid infiltration, epithelial ulceration, periodontitis, mucositis, infection, salivary glands, or blood (2, 8). Saliva-only case-control differences therefore cannot define tumor immunophenotype, predict checkpoint-inhibitor response, or establish tumor origin.

We focus on immune-related proteins, cytokines, EVs, and coding and non-coding RNAs; tumor-derived DNA is included only to distinguish tumor detection from immune readout, not to catalogue salivary omics. A three-tier framework grades tissue-saliva evidence. Level I requires quantitative same-patient concordance at the molecular, pathway, or cellular level. Level II links saliva to serum, pathology, source-enriched EVs, stage, treatment, or outcome without direct tissue correlation; Level III comprises saliva-only case-control differences. Level II support is labeled P, paired tissue-saliva presence without quantitative concordance; B, paired biofluid; S, source-enriched; L, longitudinal; or M, model-derived. This hierarchy structures the evidence summary and translational validation framework.

This focused narrative review used a targeted PubMed/MEDLINE search updated through 1 September 2026, supplemented by backward and forward citation screening. Search terms combined OSCC and saliva with cytokines, chemokines, EVs, miRNAs, lncRNAs, circRNAs, cfRNA, and ctDNA. We prioritized human OSCC studies reporting cohort size, diagnostic indices, paired compartments, longitudinal kinetics, or immune mechanisms; HNSCC studies were retained only for relevant context. The synthesis is representative rather than systematic.

## 2 Biological routes linking the tumor immune microenvironment to saliva

OSCC can affect saliva through several routes. Malignant epithelial, immune, and stromal cells can secrete mediators onto ulcerated surfaces; apoptosis, necrosis, and shedding release intracellular contents, while EVs can protect membrane proteins and RNA (2, 9). Interstitial and blood-borne components may cross mucosal or glandular barriers, and gingival crevicular fluid contributes leukocyte- and plasma-derived proteins during periodontal inflammation (8, 10). One study evaluated IL-1α, IL-6, IL-8, and TNF-α in OSCC/OPMD tissue and saliva, but different compartment sample sizes and the absence of individual-level correlation precluded source attribution (11). Spatial transcriptomics links glycolytic OSCC regions to inflammatory cancer-associated fibroblasts, CXCL12, Treg recruitment, and TGF-β-rich immunosuppression (12). Single-cell studies of HNSCC and post-treatment OSCC identify malignant subclones, macrophage states, fibroblast programs, exhausted lymphocytes, and cytokine pathways masked in bulk data (13, 14). OSCC analyses also show heterogeneous CD4, CD8, and B-cell states (15). These tissue findings nominate pathways but do not show that their products dominate saliva.

Concordance requires more than parallel group-mean changes. Strong evidence would show that same-patient saliva tracks tissue abundance or producer-cell state, falls after complete resection, and rises with molecular or clinical recurrence. Pathway-level agreement may be more realistic than one-to-one molecular matching: a multi-source cytokine might contribute to a salivary myeloid-inflammation panel aligned with a spatial myeloid niche. OSCC spatial studies support incorporating cellular organization into candidate interpretation (4, 12).

Sampling compartment also shapes concordance. Lesion-proximal saliva may enrich surface signals but is difficult to standardize across tumor sites. Whole saliva is more reproducible and convenient but mixes glandular, periodontal, microbial, and systemic inputs (5, 8). Studies should compare both compartments, record lesion-to-sampling distance, and report molecular, pathway, and cellular concordance separately rather than as one coefficient.

Salivary dysbiosis co-occurs with altered cytokine profiles in OSCC (16), but may reflect tumor-driven ecological change, pre-existing periodontitis, or both. **Figure 1** therefore depicts testable routes and competing sources rather than a single dilution pathway.

**Figure 1:**
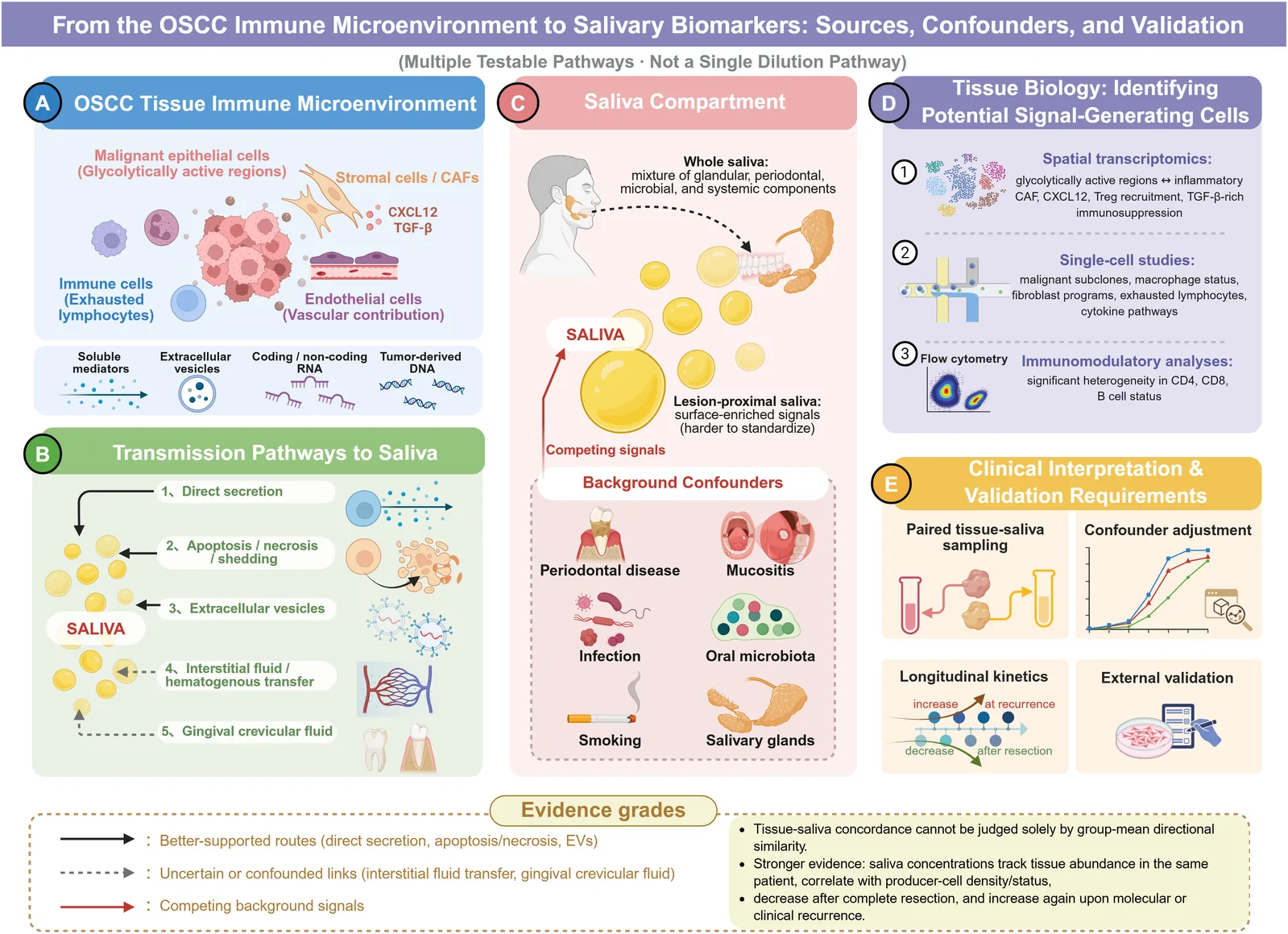
From the OSCC immune microenvironment to salivary biomarkers: sources, confounders, and validation. **(A)** Cellular sources and candidate molecular signals in the OSCC immune microenvironment. **(B)** Potential routes of signal transmission to saliva. **(C)** Salivary compartments and competing background sources. **(D)** Tissue-based approaches to identifying potential signal-generating cells. **(E)** Clinical interpretation and validation through paired tissue-saliva sampling, confounder adjustment, longitudinal assessment, and external validation.

## 3 Evidence for saliva–tissue immune concordance in OSCC

### 3.1 Soluble cytokines and chemokines

IL-8/CXCL8 and IL-6 are the most reproducible salivary cytokine candidates (7), and independent OSCC cohorts have also reported elevated IL-1β and IL-8, including studies with OPMD controls (17, 18). In an Indian cohort comprising 58 patients with active OSCC (31 early-stage and 27 late-stage), 30 patients with OPMD, 29 post-treatment patients, and 42 healthy controls, AUCs for all-stage OSCC versus controls were 0.772 for IL-1β and 0.703 for IL-8; late-stage AUCs were 0.902 and 0.762 (17). A Sri Lankan cohort of 37 OSCC cases, 30 oral epithelial dysplasia cases, and 30 controls reported AUCs of 0.978 for IL-8 (96.9% sensitivity, 96.7% specificity), 0.856 for IL-6 (75.6%, 96.6%), and 0.724 for IL-1β (64.0%, 84.0%) (18). These estimates are cohort-specific and lack external validation. Comparison of IL-6 with chronic periodontitis and OPMD controls improves assessment of inflammatory specificity (19), but neither ROC discrimination nor relevant controls establish tissue concordance; this evidence remains Level III.

Chemokine evidence is thinner. Salivary CCL20 discriminated OSCC from controls and was elevated in tissue and saliva, but no patient-level cross-compartment correlation was reported (20). IL-17, but not IL-10, differed between groups in another panel, illustrating within-panel inconsistency (21). Diabetes-stratified TNF-α studies show that systemic inflammation can distort interpretation (22). TGF-β is biologically plausible in OSCC matrix remodeling and immune exclusion (2, 12), yet salivary evidence is sparse and measurement is vulnerable to pre-analytical and tissue-injury effects (8). Without paired validation, these mediators cannot serve as surrogates for CD8 infiltration, Treg enrichment, or PD-L1 status.

Longitudinal IL-6 trajectories after surgery and adjuvant therapy provide stronger, but indirect, evidence (23). A postoperative decrease is compatible with tumor relevance, whereas chemoradiotherapy-related oral injury and changes in salivary volume and growth factors can alter the salivary milieu independently of tumor burden (24). Unless IL-6 changes track residual or recurrent disease and are separable from treatment injury, they remain Level II evidence.

### 3.2 Extracellular vesicles and immune-related RNA cargo

EV cargo may be more stable and capture markers can provide limited source information (9, 25). In paired saliva and serum from 40 OSCC patients and 40 controls, salivary exosomal TNF-α mRNA achieved an AUC of 0.88 (90% sensitivity, 80% specificity), OAZ1 an AUC of 0.87 (77.5%, 87.5%), and their combination an AUC of 0.89 (80%, 90%) (26). This is paired-biofluid, not paired-tissue, evidence. Salivary cfRNA separated OSCC, benign tumors, and controls and computationally inferred more neutrophils and fewer lymphocytes, while plasma lacked an OSCC-specific RNA signal (27); such deconvolution is hypothesis-generating, not a tissue leukocyte measurement. Salivary EpCAM-positive tumor-associated and CD45-positive immune-cell PD-L1 EVs showed different immunotherapy trajectories (25), and another method detected two EV subsets associated with T stage (28). Source enrichment and clinical association support Level II, but concordance with tissue PD-L1, immune-cell state, or EV release remains untested.

Salivary miR-21, miR-155, miR-375, and other panels are associated with OSCC or OPMD (29, 30), while mRNA panels and individual transcripts, including IL-6 mRNA, provide additional classification routes (31, 32). Candidate selection, normalization, hemolysis or cellular contamination, and thresholds vary materially (33). A salivary-EV systematic review found promising detection signals but small cohorts and inconsistent isolation and reporting (6). MISEV2023 therefore emphasizes standardized nomenclature, separation controls, particle characterization, and transparent reporting, especially because bacterial vesicles, lipoproteins, mucins, and debris may co-separate from saliva (9).

### 3.3 Secreted non-coding RNAs and cytokine-linked immune programs

Secreted ncRNAs require separate evaluation because salivary OSCC studies predominantly examine miRNAs, whereas lncRNAs and circRNAs remain less mature (33, 34). Recurrent candidates include miR-21, miR-155, and miR-375 (29, 30), and miR-21 has been measured in both OSCC tissue and liquid compartments (35). However, abundance and direction may vary among whole saliva, cell-free supernatant, oral rinse, and purified EVs. Detection in tissue and saliva does not by itself prove secretion or tumor origin, because exfoliated epithelium, leukocytes, blood, and non-tumor oral cells can contribute the same RNA (9, 33). The sampled fraction is therefore a biological variable, not merely a technical detail.

Representative clinical studies nevertheless show diagnostic potential. Park et al. validated salivary miR-125a and miR-200a in 50 OSCC cases and 50 matched controls (36). A meta-analysis of 16 articles (25 study units; 2, 562 participants) reported sensitivity 0.78, specificity 0.82, and AUC 0.91, but pooled blood and salivary miRNAs (37). In 89 OSCC cases and 58 controls, a three-miRNA signature achieved a cross-validated AUC of 0.923, with 85.4% sensitivity and 85.1% specificity. MiR-423-5p also declined in 15 paired postoperative samples (38). Across two cohorts totaling 83 cases and 54 controls, miR-30c-5p achieved AUC 0.82, 86% sensitivity, and 74% specificity, although tissue and saliva changed in discordant directions (39). A four-miRNA panel in 31 cases and 31 controls achieved AUC 0.954, but validation was internal (40). These indices support diagnostic development, but case-control and internally validated designs do not establish tumor-to-saliva transport or immune concordance.

Paired tissue-saliva data remain rare. In 130 patients, miR-21 accuracy for nodal metastasis was 65.0-71.54% in saliva and 69.0-81.54% in tissue, without an individual-level cross-compartment correlation (41). Parallel accuracy estimates and detection in both compartments do not show that higher tumor expression produces higher salivary concentration. Paired collection alone therefore supports Level II-P, not Level I.

Mechanistic studies provide a separate line of evidence. OSCC EV miR-21 activated monocyte NF-κB and increased IL-6, CCL2, PGE2, and MMP9 (42), whereas exosomal miR-29a-3p promoted M2 polarization through SOCS1/STAT6 (43). These findings link secreted miRNAs to cytokine and myeloid programs, but do not show that salivary abundance tracks the corresponding tumor pathway in the same patient. Clinical studies should therefore quantify ncRNAs and cytokines in the same saliva aliquot. These measurements should be compared with spatial tumor STAT3/NF-κB activity and macrophage phenotype.

Evidence for lncRNAs is more limited. A pilot study analyzed 20 tissue specimens (16 tumors and 4 matched adjacent non-tumor specimens) and saliva from nine patients. MALAT1 was detected in 9/9 saliva samples and HOTAIR in 5/9; metastatic tissues also showed higher HOTAIR, NEAT1, and UCA1, but no quantitative tissue-saliva correlation or ROC analysis was performed (34). Moreover, pelleted whole-saliva RNA cannot distinguish secreted from cellular material. In experimental models, cancer-stem-cell EV UCA1 promoted IL-10-positive M2 macrophages and suppressed CD4-positive T-cell proliferation and IFN-γ through the miR-134/LAMC2/PI3K-AKT axis (44). Paired presence therefore supports Level II-P, while the UCA1 experiment supplies mechanistic plausibility; neither establishes a salivary immune surrogate.

Case-control studies add diagnostic, not tissue-concordance, evidence. Salivary MALAT1 in 20 cases and 20 controls yielded 95% sensitivity and 90% specificity at a cut-off of 2.24 (45). In 59 cases and 43 controls, absent salivary XIST was associated with OSCC (AUC 0.73; odds ratio 19.556), with sex-specific expression (46). Neither study measured matched tumor lncRNA or cytokine activity. The inverse MALAT1-miR-124 association is compatible with, but does not prove, a sponge mechanism.

CircRNAs provide longitudinal support. In 93 OSCC cases, 85 healthy controls, and 70 patients with leukoplakia, followed by validation in 90 cases and 82 controls, hsa_circ_0001874 plus hsa_circ_0001971 achieved AUC 0.922 and both declined in 25 paired samples one month after resection (47). Postoperative decline supports tumor relevance but may also reflect resection-related changes. Without tumor tissue or cytokine endpoints, this remains Level II-L evidence.

Overall, salivary detection, limited tissue-saliva or longitudinal support, and experimental cytokine/TME regulation are distinct evidentiary claims and must not be conflated. No salivary ncRNA candidate reaches Level I because no study demonstrates a same-patient quantitative association between salivary ncRNA and a defined tissue cytokine or immune-cell state after adjustment for oral inflammation.

### 3.4 Salivary tumor-derived DNA: strong tumor traceability, limited immune interpretation

Salivary tumor-derived DNA illustrates strong tumor traceability without immune concordance. Among 93 patients with HNSCC, tumor DNA was detected in saliva from all 46 oral-cavity cancers and from 76% across all head and neck sites; among 47 patients providing both saliva and plasma, either biofluid detected tumor DNA in 96% (48). Among 121 treatment-naive oral cavity squamous cell carcinoma cases with matched tumor and preoperative saline oral-rinse samples, 106 tumors harbored panel-detectable mutations; matched variants were found in 99 of these 106 pairs (93.4%), including 88% of early-stage cases (49). Tumor-derived DNA signals primarily reflect shedding, lesion contact, burden, and cell death rather than immune state (48, 49). They are tumor-origin evidence and candidates for minimal residual disease monitoring, not validated immune surrogates. Collection device, preservative, processing delay, and cellular fraction (50), together with sequencing depth and the variant threshold (49), should be reported.

### 3.5 Lessons from non-immune paired omics studies

Paired metabolomics is a methodological precedent, not immune evidence. An OSCC study combined salivary metabolomics with tissue mass-spectrometry imaging to identify disease-related patterns (51). The same sequence can guide immunobiomarker studies: define spatial tissue states first, then test whether a simplified salivary panel preserves a traceable signal. By contrast, OSCC EV mtDNA–PD-L1 data remain mechanistic rather than salivary evidence (52).

### 3.6 Overall evidence assessment and why Level I remains unfilled

No salivary immune marker currently meets Level I by linking an individual salivary measurement to the corresponding tumor immune state. Soluble cytokines are mainly Level III, whereas longitudinal kinetics, paired biofluids, source-enriched EVs, model-derived cell fractions, and limited paired tissue-saliva presence provide distinct forms of Level II support. Assigning a single highest level plus a modifier avoids uninformative “II-III” ranges. Diagnostic discrimination does not confirm origin, and saliva cannot replace tissue immunoprofiling; adjunctive triage, serial monitoring, and recurrence surveillance require prospective validation against a tissue-established baseline.

Level I is absent for structural reasons. Tissue and saliva are seldom collected simultaneously; biopsy material may be inadequate for spatial immune assays; ulceration and oral inflammation can overwhelm tumor-niche signals; and whole saliva integrates glands, mucosa, periodontal tissues, microbes, and blood (8, 16). A bulk-tissue mean may also miss spatial heterogeneity (4, 12), while saliva varies over hours and tissue is usually sampled once. Normalization, low abundance, multiplicity, and small cohorts further limit within-patient estimates (6, 33).

Level I denotes direct quantitative same-patient tissue-saliva concordance at the molecular, pathway, or cellular level. Studies should predefine the tissue immune state, collect tumor and saliva closely in time, and test individual-level associations with a locked assay adjusted for periodontal disease, ulceration, flow rate, and blood contamination. Adjacent mucosa or plasma controls, perioperative or recurrence kinetics, and multicenter replication strengthen source attribution and validation maturity but are not Level I prerequisites. **Table 1** presents the assignments; cohort sizes and diagnostic indices remain in the narrative.

**Table 1:** Candidate salivary immune biomarkers in oscc and the strength of evidence linking them to the tumor microenvironment.

Biomarker or class	Proposed tissue source	Evidence in saliva	Direct tissue–saliva concordance	Clinical association	Major confounders	Evidence level	Key validation requirement
IL-6	Tumor cells, myeloid cells, CAFs	Longitudinal treatment trajectory; tissue/saliva group-level comparison (11, 23)	Same-study tissue/saliva only; no robust patient-level correlation	OSCC status; longitudinal treatment change	Periodontitis, diabetes, wound healing, RT	II-L	Paired spatial tissue IL-6/JAK–STAT state and perioperative saliva
IL-8/CXCL8	Tumor epithelium, neutrophils, myeloid cells	Replicated case-control discrimination; group-level tissue expression (11, 17)	Parallel tissue and salivary elevation; correlation unproven	OSCC/OPMD discrimination	Periodontitis, infection, ulceration, smoking	III	Same-patient tissue expression, neutrophil mapping, oral-inflammatory controls
IL-1β	Myeloid cells, damaged epithelium	Salivary case-control difference (18)	No robust direct concordance	Case–control discrimination	Periodontitis, mucositis, sample blood	III	Tumor/adjacent tissue pairing and periodontal adjustment
TNF-α	Myeloid and epithelial cells	Paired saliva-serum EV-mRNA support; salivary protein case-control data (22, 26)	No robust direct concordance	OSCC status; affected by diabetes	Diabetes, tobacco, periodontitis	II-B	Protein plus EV-RNA concordance with tissue myeloid states
IL-10	Tregs, macrophages, tumor cells	No significant between-group difference in one study (21)	Not established	No significant OSCC/OPMD group difference in the cited study	Oral inflammation, systemic immune disease	III	Pair saliva with tissue Treg/macrophage abundance
TGF-β	CAFs, Tregs, tumor cells	No direct salivary evidence in the cited study; tissue mechanistic plausibility (12)	Not established	Biological plausibility more than salivary validation	Platelets, latent activation, wound healing	III	Active/total assay plus spatial CAF–Treg tissue state
Soluble/EV PD-L1	Tumor and immune-cell EVs	Source-enriched salivary EV capture with longitudinal treatment trajectories (25)	Tissue PD-L1 concordance not yet robust	Response trajectories in an early study	EV capture bias, oral immune cells	II-SL	Paired tissue CPS/spatial PD-L1 and serial EV subtypes
EV immune mRNAs	Tumor, immune and stromal EVs	Paired saliva-serum exosomal mRNA study (26)	Paired saliva–serum, not tissue	Diagnosis, grade, nodal status	Isolation purity, bacterial EVs, cellular RNA	II-B	Tissue source mapping and standardized EV recovery
miRNA panels	Tumor and non-tumor oral cells	Limited paired tissue-fluid presence for selected miRNAs; most panels case-control only. Longitudinal miR-423-5p decline and paired tissue-saliva miR-21 sampling provide additional indirect support (38, 41).	Same-patient tumor and saliva were collected for miR-21, but individual-level cross-compartment concordance was not demonstrated (41).	OSCC/OPMD classification. Nodal status, disease-free survival, and postoperative change were also evaluated (38, 41).	Normalization, hemolysis, inflammation	II-PL	Locked small panel with tissue directionality and external validation. Include matched salivary cytokine readouts and spatial tissue immune states.
cfRNA signatures	Shedding, cell death, secreted RNP/EV cargo	Model-derived immune-cell fractions; no paired tissue reference (27)	Immune deconvolution only; no tissue match	OSCC vs benign/healthy groups	Cell lysis, oral leukocytes, RNA stability	II-M	Paired tissue single-cell reference and prospective replication
Salivary lncRNAs	Tumor cells and CSC-derived EVs (44)	MALAT1 detected in 9/9 and HOTAIR in 5/9 OSCC saliva samples in one pilot paired-compartment study (34). Additional case-control studies support salivary MALAT1 and XIST as diagnostic candidates (45, 46).	Same patients provided tissue and saliva, but no quantitative concordance analysis	Exploratory metastasis association; no validated ROC threshold. MALAT1 was associated with nodal involvement/metastasis; XIST was sex-specific.	Low abundance, cellular contamination, normalization, RNA stability	II-P	Same-patient EV/whole-saliva lncRNA plus cytokine panel matched to spatial macrophage and T-cell states. External validation and matched tissue-cytokine measurements remain required.

Evidence levels: I, direct quantitative same-patient tissue-saliva concordance; II, indirect clinicopathological, paired-compartment, source-enriched, model-derived, or longitudinal support; III, salivary case-control difference only. Level II modifiers identify the supporting design: P, paired tissue-saliva presence without quantitative concordance; B, paired biofluid; S, source-enriched; L, longitudinal; M, model-derived. No salivary immune marker currently fulfills Level I. CAF, cancer-associated fibroblast; CSC, cancer stem cell; EV, extracellular vesicle; OPMD, oral potentially malignant disorder; RT, radiotherapy.

## 4 Why saliva may fail to mirror tumor immunity

Biological confounding represents a second immune ecosystem. Oral health, occult bleeding, and collection conditions influence salivary IL-1β, IL-6, IL-8, and TNF-α (8). cfDNA recovery varies with device, preservative, processing delay, and cell lysis (50); cfRNA needs comparable controls, and the cited saliva-blood study found no OSCC-specific plasma RNA signal (27). A periodontitis meta-analysis identified salivary IL-1β and IL-6 as discriminative markers (10); panels developed against exceptionally healthy controls may therefore fail in older smokers or patients with gingivitis, OPMD, diabetes, or benign ulceration.

Lesion contact and ulceration may alter recovery of tumor-derived material, but this effect has not been consistently quantified across oral subsites (48, 49). Infection, necrosis, hemorrhage, tumor volume, nodal status, and gland function can change signal intensity without changing immunophenotype (8, 28). Saliva also combines major- and minor-gland secretions, mucosal exudate, and gingival crevicular fluid. Normalization to total protein may reduce dilution effects yet introduce bias when total protein secretion changes (8, 9).

Chemoradiotherapy can reduce stimulated saliva volume and alter salivary growth-factor profiles (24). Without longitudinal controls, such treatment effects should not be interpreted as residual OSCC.

Pre-analytical variation can materially change effects (8, 50). Stimulation changes flow and glandular contribution; whole and lesion-proximal saliva also differ. Studies should predefine eating, brushing, rinsing, circadian phase, collection time, centrifugation, storage temperature, freeze-thaw cycles, and standardization (8, 50). Protein assays, qPCR, sequencing, and microfluidic EV platforms differ in detection limits and matrix effects (9, 33). After collection, proteases cleave cytokines, nucleases alter free RNA, and residual cells release transcripts during processing delays (8, 33); mucins and abundant proteins may obscure low-level targets or impair capture. EV particle counts, total protein, and target-positive events measure different phenomena, and particle normalization may conceal disease-related vesicle release (9). Reports should give raw concentrations, flow rates, total protein or particle counts, recovery controls, and normalization rationale. Batch, well, operator, and storage time should be balanced across groups, with blinded analysts (8, 9). Technical reproducibility cannot correct systematic pre-analytical imbalance.

Clinical labels require equal care. Oral OSCC should not be pooled with all oral cancers, HNSCC, or HPV-related oropharyngeal disease. Analyses should stratify oral subsite, stage, recurrence, treatment, exposures, periodontal status, and OPMD history; otherwise cohort confounding may appear as biomarker failure (5).

## 5 A translational validation framework

Validation should begin with a predefined clinical use: detection, OPMD risk, nodal disease, minimal residual disease, recurrence, or treatment response. Each claim requires different cohorts and endpoints, and current prognostic evidence remains heterogeneous (5, 53). At one visit, exploratory cohorts should collect tumor, adjacent mucosa, whole and, where feasible, lesion-adjacent saliva, and plasma. Sampling should occur within a narrow time window, with spatially separated tumor regions when feasible. Tissue state should be defined by multiplex or spatial assays (4, 12), including single-cell assessment of treatment-related T-cell, myeloid, and fibroblast states (54) and attention to endothelial, matrix, and immune features obscured by bulk averages (55, 56). Candidate retention should require association with these predefined tissue states, not merely case-control separation. Controls should include healthy participants, periodontitis or other oral inflammation, OPMD, OSCC, and preferably post-treatment patients without disease. Models should adjust for age, sex, subsite, tobacco, alcohol or areca nut, diabetes, oral hygiene, periodontal status, ulceration, stage, medication, and salivary flow (5, 10). Markers lost after adjustment may reflect local inflammation, but should not be called tumor-immune specific.

Longitudinal sampling should include baseline, post-resection, post-adjuvant treatment, surveillance, and recurrence time points (23, 38). Cross-sectional detection of PDCD4 and miR-21 in tissue and fluids does not establish kinetics (35). Likewise, exosomal PD-L1-mediated tumor-myeloid communication in HNSCC (57) and EV PD-L1/mitochondrial-DNA immunosuppression in OSCC (52) are mechanistic evidence, not proof that salivary EV measurements quantitatively mirror tissue state. The decisive test is whether a salivary trajectory follows paired tissue change or objective recurrence after separating effects of surgery, mucositis, infection, and salivary-gland injury. Sampling intervals, treatment exposure, and oral inflammatory events must be prospectively recorded.

Assays should progress from discovery to locked, small panels with prespecified precision, linearity, quantitation limits, stability, and failure rules. EV studies require recovery and purity controls, and RNA studies require extraction, contamination, and normalization specifications (9, 33); cytokine assays require matrix-spiking and dilution checks (8). Raw saliva volume or flow and total protein or particle counts should be reported rather than concealed by one normalization scheme. Secreted ncRNAs and predicted cytokine outputs should be measured in the same aliquot and analyzed against paired spatial tissue pathways. Thresholds should be fixed before external validation and compared with examination, imaging, histopathology, stage, PD-L1 combined positive score, and routine inflammatory markers (5, 7).

Analysis plans should separate feature selection from performance estimation, prespecify missing-data and below-quantitation handling (58), and base sample size on intended endpoints and event rates (58, 59). Longitudinal models should emphasize within-patient change while accounting for postoperative inflammation and baseline variability. Reporting should extend beyond ROC AUC to calibration, clinically acceptable false-positive rates, recurrence lead time, decision curves, net benefit, avoided biopsies, and incremental value over standard care (60). Calibration estimates and confidence intervals should accompany discrimination, and decision thresholds should be locked before external testing. Final validation should be prospective, multicenter, geographically diverse, and externally replicated, with salivary predictions compared against paired tissue and objective treatment outcomes. Independent assay reproducibility and transportability across sites should be demonstrated before clinical implementation. The goal is a biologically anchored, dynamically interpretable panel, not a universally applied diagnostic score.

## 6 Conclusions and future priorities

Saliva captures OSCC-associated inflammation, secreted ncRNAs, EVs, and tumor DNA, but each analyte supports a different inference. Tumor DNA can establish tumor-derived material without characterizing immunity. Cytokine, miRNA, lncRNA, and EV studies support disease association or mechanistic plausibility more strongly than same-patient tissue concordance. Stronger indirect designs use inflammatory controls, paired humoral measurements, source-enriched EV capture, spatial tissue phenotyping, or longitudinal sampling. Nevertheless, no salivary immune marker robustly links an individual-level measurement to a defined immune-cell or pathway state in the same tumor.

Near-term research should evaluate saliva as a complement to examination and histopathology for postoperative surveillance and treatment-response monitoring (23). Current prognostic evidence remains heterogeneous and insufficient for implementation (53). Progress requires same-patient tissue-saliva studies, control of oral inflammation, perioperative kinetics, locked methods, and multicenter validation. Negative concordance studies are also informative. Clinical value depends on whether salivary signals can be traced to tumor immunobiology, separated from oral inflammatory noise, and reproduced longitudinally across centers.
